# A Randomized, Double-Blind, Placebo-Controlled, Parallel-Group Phase 2b Trial of P2X3 Receptor Antagonist Sivopixant for Refractory or Unexplained Chronic Cough

**DOI:** 10.1007/s00408-022-00592-5

**Published:** 2022-12-13

**Authors:** Lorcan McGarvey, Jaclyn A. Smith, Alyn Morice, Surinder S. Birring, Kian Fan Chung, Peter V. Dicpinigaitis, Akio Niimi, Michael S. Benninger, Mandel Sher, Yuko Matsunaga, Sayaka Miyazaki, Mitsuaki Machida, Hiroyuki Ishihara, Adnan Mahmood, Juan-Carlos Gomez

**Affiliations:** 1 Queen’s University Belfast, Belfast, Northern Ireland UK; 2 University of Manchester and Manchester University NHS Foundation Trust, Manchester, UK; 3 University of Hull, Castle Hill Hospital, Hull, UK; 4 Centre for Human & Applied Physiological Sciences, School of Basic & Medical Biosciences, Faculty of Life Sciences & Medicine, King’s College London, London, UK; 5 National Heart & Lung Institute, Imperial College London & Royal Brompton and Harefield Hospitals, London, UK; 6 Albert Einstein College of Medicine, Montefiore Medical Center, Division of Critical Care Medicine, Bronx, NY USA; 7 Department of Respiratory Medicine, Allergy and Clinical Immunology, Nagoya City University, Nagoya, Japan; 8 Head and Neck Institute, The Cleveland Clinic, Cleveland, OH USA; 9 University of South Florida, Tampa, FL USA; 10 Shionogi Inc., Florham Park, NJ USA; 11 Shionogi & Co., Ltd., Osaka, Japan; 12 Shionogi B.V., 33 Kingsway, London, WC2B 6UF UK

**Keywords:** Chronic cough, Cough frequency, P2X3 receptor antagonist, Phase 2b trial, Sivopixant

## Abstract

**Introduction:**

To determine the optimal dose of sivopixant, a highly selective P2X3 receptor antagonist, for refractory or unexplained chronic cough (RCC/UCC).

**Methods:**

In this phase 2b, randomized, double-blind, placebo-controlled, parallel-group, multicenter trial, patients received sivopixant 50, 150, or 300 mg or placebo once daily for 4 weeks. The primary endpoint was a change from baseline in 24-h cough frequency (coughs/h) with sivopixant vs placebo.

**Results:**

Overall, 390/406 randomized patients completed the study. Placebo-adjusted changes in hourly cough count over 24 h were 13.17% (*P* = 0.3532), − 1.77% (*P* = 0.8935), and − 12.47% (*P* = 0.3241) and in cough severity (visual analog scale) were 1.75 mm (*P* = 0.5854), − 1.21 mm (*P* = 0.7056), and − 6.55 mm (*P* = 0.0433) with sivopixant 50, 150, and 300 mg, respectively. Placebo-adjusted changes from baseline in Leicester Cough Questionnaire total scores were − 0.37 (*P* = 0.4207), − 0.07 (*P* = 0.8806), and 0.69 (*P* = 0.1473) with sivopixant 50, 150, and 300 mg, respectively. Additionally, 61.3%, 78.3%, 86.8%, and 71.4% of patients receiving sivopixant 50, 150, and 300 mg and placebo, respectively, reported any improvements in Patient Global Impression of Change. The incidence of treatment-emergent adverse events (TEAEs) was 25.7%, 32.0%, 49.0%, and 20.6% in sivopixant 50, 150, and 300 mg and placebo groups, respectively; all TEAEs in the sivopixant group were mild-to-moderate.

**Conclusion:**

Sivopixant did not demonstrate a statistically significant difference vs placebo in change from baseline in 24-h cough frequency. The dose of 300 mg has potential for RCC/UCC, showing the greatest improvements in cough frequency and patient-reported outcomes and dose-related mild to moderate reversible taste disturbance, although further trials are needed.

**Clinical Trial Registration:**

ClinicalTrials.gov identifier NCT04110054; registered September 26, 2019.

**Supplementary Information:**

The online version contains supplementary material available at 10.1007/s00408-022-00592-5.

## Introduction

Chronic cough (CC), defined as cough lasting > 8 weeks, has an estimated global prevalence of 9.6% [1]. Refractory CC (RCC) is defined as CC that persists despite optimal treatment of associated conditions [2, 3] and unexplained CC (UCC) as CC with no known etiology despite extensive clinical assessment and therapeutic trials [3, 4]. Approximately 20–46% of patients presenting to specialist cough clinics have RCC/UCC [5, 6] and face significant physical, psychological, social, and financial burden, resulting in poor quality of life (QoL) [4, 7, 8]. Thus, a significant unmet need exists for safe and effective therapeutic agents for RCC/UCC that can be administered long-term [9, 10].

The P2X3 receptor, an adenosine triphosphate-gated ion channel primarily expressed by sensory nerves, including the airway vagal afferent nerves, has been implicated in cough reflex activation resulting in CC [11, 12]. Several randomized controlled trials (RCTs) have identified P2X3 receptor antagonists as promising therapeutic agents for CC; however, treatment with these agents has been associated with taste disturbances that may affect patients’ QoL and decrease treatment adherence [13–16]. Sivopixant (S-600918) is a potent P2X3 receptor antagonist with high selectivity for P2X3 over P2X2/3 that may offer advantages including reduced taste disturbance and once-daily (OD) dosing [17]. A phase 2a proof-of-concept crossover study in 31 patients with RCC/UCC found that treatment with sivopixant 150 mg OD for 2 weeks resulted in a − 31.6% and − 30.9% placebo-adjusted change from baseline in the daytime and 24-h average hourly cough frequency, respectively, and a significant improvement in the Leicester Cough Questionnaire (LCQ) total score without major safety concerns [18]. These data support the therapeutic effect of sivopixant. The purpose of this phase 2b study was to determine the optimal dose of sivopixant to treat adults with RCC/UCC.

## Methods

### Study Design

This multinational, multicenter, randomized, double-blind, placebo-controlled, parallel-group phase 2b trial was conducted at 112 study sites in Europe, the United States, and Japan (NCT04110054). Adults who met the inclusion criteria at screening Visit 1 (18–28 days before randomization) were enrolled and underwent 24-h cough monitoring using the VitaloJAK™ cough monitor [19]. At screening Visit 2 (1 day before randomization), patients underwent a further 24-h cough monitoring using the cough monitor. Cough count at Visit 1 was used to assess eligibility and for stratification, whereas cough count at Visit 2 was used as the baseline assessment.

Patients who continued to meet the screening criteria at Visit 3 were randomized in a 1:1:1:1 ratio (93 patients per treatment group) to receive sivopixant 50, 150, or 300 mg or placebo OD for 4 weeks (e-Fig. 1, Online Resource). Randomization was stratified by region (Europe, the United States, or Japan) and 24-h cough count at Visit 1 (≥ 30 or < 30 coughs/h).

The randomization schedule was maintained by interactive response technology until data lock. The sponsor, investigator, and all study-site personnel were blinded to treatment assignment until data lock.

Placebo tablets matched in appearance with the sivopixant 50-mg tablet, and the labeling and packaging of sivopixant and placebo were identical. Patients were followed up for safety for 2 weeks after their last dose.

### Patients

Adults aged 18–80 years with RCC/UCC for ≥ 1 year with a cough severity visual analog scale (VAS) score ≥ 40 mm at both screening Visits 1 and 2 and ≥ 10 coughs/h on the 24-h cough count recording at Visit 1 were eligible. Most patients were recruited from pulmonology/respiratory or allergy sites. Diagnosis of RCC/UCC was made by the investigators, and the sponsor did not oversee the accuracy of the diagnosis. Detailed inclusion/exclusion criteria are listed in e-Appendix 1 (Online Resource).

### Outcome Assessment and Statistical Analysis

The primary endpoint was the ratio of the number of coughs/h in 24 h at Week 4 to that at baseline. Secondary efficacy endpoints included the ratio of the number of coughs/h while awake/asleep to that at baseline and change from baseline in cough severity (VAS; 0–100 mm; 0: no cough, 100: worst cough ever) [20, 21], LCQ total score [22], and Patient Global Impression of Change (PGIC) [23], which consists of one item adapted from the Clinical Global Impressions scale [24]. Detailed definitions of the endpoints are provided in e-Appendix 2, Online Resource**.** An improvement in LCQ total score of ≥ 1.3 points identified a treatment responder [25, 26]. Safety assessments included monitoring treatment-emergent adverse events (TEAEs) at each visit, classified by System Organ Class and Preferred Terms using the Medical Dictionary for Regulatory Activities, version 23.0.

The previous phase 2a study observed a mean ± standard deviation (SD) log-transformed ratio of coughs/h in 24 h after 2 weeks of treatment to that at baseline of − 0.327 ± 0.379 with sivopixant 150 mg and − 0.160 ± 0.363 with placebo. The effect size between the placebo and sivopixant groups was assumed to be − 0.44 and the same at 4 weeks. Based on that, the sample size in the current study (80% power using a 2-sided 5% significance level) was calculated as 83 patients per group. By allowing a dropout rate of 10%, 93 patients per treatment group would be needed. Thus, the planned sample size at randomization was 372 patients.

All efficacy analyses were performed in the full analysis set (FAS), and safety analysis was performed in the safety population. The primary endpoint analysis was additionally performed in the per-protocol set (PPS) as supplemental analysis. The analysis sets have been described in e-Appendix 3 (Online Resource).

For the analysis of the primary efficacy endpoint, the ratio of the number of coughs/h in 24 h at each visit to that at baseline was transformed using the common logarithm. The primary endpoints were assessed using a mixed-effects model, with treatment group, week, and the interaction between treatment group and week as fixed effects; patient as random effect; and region and log-transformed coughs/h in 24 h at baseline as covariates. Missing data for cough frequency were not imputed, and observed data were used for the model. Baseline value was defined as the last valid assessment recorded prior to first administration of the study medication. The primary analysis summarized each of the three 2-way comparisons with placebo and presented the estimate for the difference in treatment effect at Week 4 and 95% confidence interval (CI) for this difference. The *P* value was generated based on the model described above; *P* < 0.05 was considered statistically significant. Multiplicity adjustment was not performed because this study was exploratory with multiple doses and not a confirmatory study.

In the proof-of-concept study, four patients with baseline hourly cough count values < 10 were included. However, the placebo-adjusted change in cough count was highly variable in this subgroup, and for this reason, patients with cough count < 10 at Visit 1 were excluded from this phase 2b trial. However, we did undertake a post hoc subgroup analysis for the primary and some secondary endpoints in patients with ≥ 10 and < 10 coughs/h in 24 h at Visit 2. All analyses and tabulations were performed using SAS, version 9.2 or higher.

## Results

### Patients

**Fig. 1:**
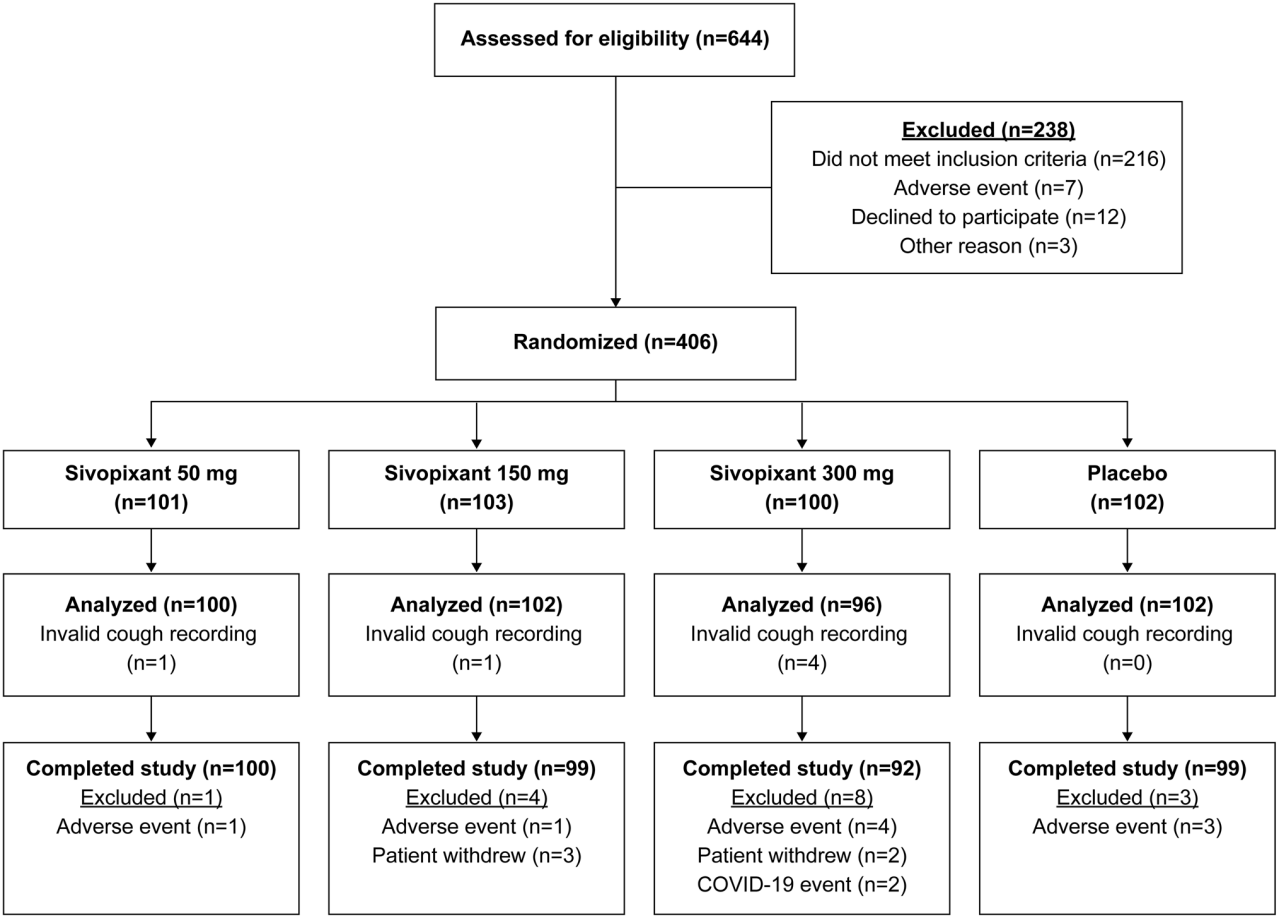
Study CONSORT flow diagram. For the analyzed population: invalid cough count recording is postbaseline. Invalid cough count: cough count recording was considered to have failed for any reason. *CONSORT* Consolidated Standards of Reporting Trials, *COVID-19* coronavirus disease 2019

Between January and December 2020, 644 patients were screened and 406 were enrolled (Fig. 1). Six patients were excluded due to absence of postbaseline cough recordings, resulting in 400 patients in the FAS. Most patients (96.1%; 390/406) completed the study. Reasons for premature study discontinuation included adverse events (AEs; *n* = 9), patient withdrawal (*n* = 5), and COVID-19 event (*n* = 2) (Fig. 1).

Baseline demographic and disease characteristics were balanced between the treatment groups (Table 1). Mean (SD) age of the total population was 57.0 (11.9) years, and most patients were female (73.8%). Geometric mean hourly cough count in 24 h was similar between the groups at baseline (sivopixant 50 mg: 24.36; sivopixant 150 mg: 24.68; sivopixant 300 mg: 26.19; placebo: 24.47).

**Table 1:** Patient demographics and baseline characteristics (FAS)

Demographic/characteristic	Sivopixant			Placebo	Total
	50 mg *N* = 100	150 mg *N* = 102	300 mg *N* = 96	*N* = 102	*N* = 400
Age (years), mean (SD)	58.7 (11.2)	57.2 (12.5)	56.1 (12.4)	56.1 (11.5)	57.0 (11.9)
Age category (years), *n* (%)					
≥ 18 to < 45	8 (8.0)	14 (13.7)	15 (15.6)	18 (17.6)	55 (13.8)
≥ 45 to < 65	55 (55.0)	55 (53.9)	55 (57.3)	60 (58.8)	225 (56.3)
≥ 65 to ≤ 80	37 (37.0)	33 (32.4)	26 (27.1)	24 (23.5)	120 (30.0)
Female, *n* (%)	78 (78.0)	75 (73.5)	65 (67.7)	77 (75.5)	295 (73.8)
Race, *n* (%)					
White	71 (71.0)	76 (74.5)	69 (71.9)	72 (70.6)	288 (72.0)
Black or African American	3 (3.0)	1 (1.0)	1 (1.0)	4 (3.9)	9 (2.3)
Asian	25 (25.0)	25 (24.5)	25 (26.0)	22 (21.6)	97 (24.3)
American Indian or Alaska Native	0	0	0	2 (2.0)	2 (0.5)
Other	1 (1.0)	0	1 (1.0)	2 (2.0)	4 (1.0)
Weight (kg)					
Mean (SD)	76.3 (19.9)	72.8 (17.8)	77.0 (18.4)	77.2 (21.5)	75.8 (19.5)
BMI, (kg/m^2^), mean (SD)	27.9 (6.2)	26.8 (5.4)	28.2 (6.4)	28.5 (7.6)	27.8 (6.5)
Region, *n* (%)					
Europe	41 (41.0)	41 (40.2)	38 (39.6)	41 (40.2)	161 (40.3)
United States	37 (37.0)	38 (37.3)	36 (37.5)	38 (37.3)	149 (37.3)
Japan	22 (22.0)	23 (22.5)	22 (22.9)	23 (22.5)	90 (22.5)
Hourly cough count in 24 h					
Mean (SD)	30.40 (22.92)	40.95 (64.68)	36.21 (37.91)	33.85 (34.60)	35.36 (43.00)
Geometric mean (95% CI)^a^	24.36 (21.31, 27.85)	24.68 (20.42, 29.82)	26.19 (22.45, 30.55)	24.47 (20.95, 28.60)	24.90 (23.01, 26.94)
Categories of hourly cough count in 24 h					
< 10, *n* (%)	13 (13.0)	13 (12.7)	10 (10.4)	10 (9.8)	46 (11.5)
Mean (SD)	7.84 (1.70)	6.79 (2.86)	8.08 (1.30)	6.19 (2.34)	7.24 (2.22)
Geometric mean (95% CI)^a^	7.66 (6.68, 8.78)	5.75 (3.66, 9.03)	7.98 (7.06, 9.01)	5.79 (4.40, 7.64)	6.71 (5.83, 77.71)
≥ 10, *n* (%)	87 (87.0)	89 (87.3)	86 (89.6)	92 (90.2)	354 (88. 5)
Mean (SD)	33.77 (22.72)	45.94 (67.85)	39.48 (38.75)	36.86 (35.14)	39.02 (44.42)
Geometric mean (95% CI)^a^	28.96 (25.85, 32.44)	30.53 (25.83, 36.09)	30.07 (26.01, 34.76)	28.62 (24.99, 32.79)	29.53 (27.53, 31.67)
Duration of CC, months					
*n*	97	102	93	95	387
Mean (SD)	138.8 (123.1)	131.7 (112.7)	118.9 (129.1)	112.7 (101.4)	125.7 (116.9)
UCC, *n* (%)	40 (40. 0)	33 (32.4)	43 (44.8)	42 (41.2)	158 (39.5)
LCQ total score, mean (SD)	10.5 (3.2)	10.5 (3.0)	10.6 (3.1)	11.0 (3.2)	10.6 (3.1)
Weekly severity of cough (VAS), mean (SD)	74.8 (14.0)	73.6 (13.8)	72.6 (15.3)	71.6 (14.5)	73.1 (14.4)

*BMI* body mass index, *CC* chronic cough, *CI* confidence interval, *FAS* full analysis set, *LCQ* Leicester Cough Questionnaire, *SD* standard deviation, *UCC* unexplained chronic cough, *VAS* visual analog scale

^a^95% CI is the 95% CI for the geometric mean

### Change in Hourly Cough Count

**Fig. 2:**
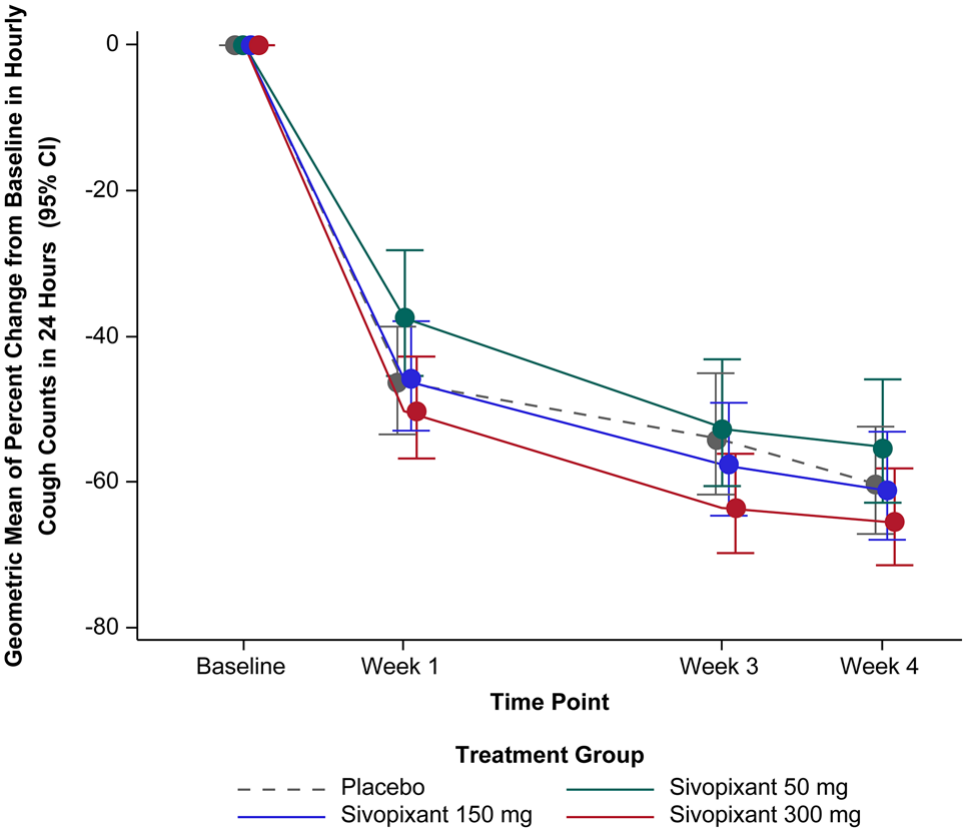
Geometric mean of percent change in hourly cough counts in 24 h from baseline to weeks 1 to 4 (FAS). Treatment effects and their 95% CIs plotted are based on a mixed-effects model for the log-transformed ratio of the number of coughs/h in 24 h at Weeks 1, 3 and 4, with treatment group, week, and interaction between treatment group and week as fixed effects; patient as random effect; and region and the log-transformed coughs/h in 24 h at baseline as covariates. Modeled estimates are presented as geometric mean of percent change from baseline. *CI* confidence interval, *FAS* full analysis set

Percent change from baseline in hourly cough counts in 24 h following 4 weeks of treatment was − 55.16%, − 61.08%, and − 65.32% with sivopixant 50, 150, and 300 mg, respectively, and − 60.38% with placebo (Fig. 2).

In the FAS, placebo-adjusted changes from baseline in hourly cough counts in 24 h after 4 weeks of treatment were 13.17%, − 1.77%, and − 12.47% with sivopixant 50, 150, and 300 mg, respectively. No significant differences were observed across the sivopixant groups and placebo group (*P* > 0.05) (Table 2).

**Table 2:** Percent change in hourly cough counts in 24 h from baseline at Week 1, Week 3, and Week 4 (FAS)

Modeled estimates^a^	Sivopixant			Placebo
	50 mg *N* = 100	150 mg *N* = 102	300 mg *N* = 96	*N* = 102
Week 1	*n* = 98	*n* = 94	*n* = 91	*n* = 101
Change, % (95% CI)	− 37.34 (− 45.34, − 28.17)	− 45.93 (− 52.85, − 37.98)	− 50.22 (− 56.74, − 42.73)	− 46.37 (− 53.13, − 38.64)
Placebo-adjusted change (95% CI)	16.85 (− 3.38, 41.32)	0.83 (− 16.67, 22.02)	− 7.18 (− 23.47, 12.59)	–
*P* value^b^	0.1082	0.9318	0.4484	–
Week 3	*n* = 93	*n* = 94	*n* = 90	*n* = 96
Change, % (95% CI)	− 52.56 (− 60.46, − 43.08)	− 57.50 (− 64.53, − 49.07)	− 63.49 (− 69.69, − 56.02)	− 54.09 (− 61.66, − 45.03)
Placebo-adjusted change (95% CI)	3.35 (− 19.90, 33.35)	− 7.42 (− 28.19, 19.37)	− 20.46 (− 38.54, 2.93)	–
*P* value^b^	0.7996	0.5512	0.0816	–
Week 4	*n* = 92	*n* = 89	*n* = 87	*n* = 95
Change, % (95% CI)	− 55.16 (− 62.80, − 45.94)	− 61.08 (− 67. 71, − 53.08)	− 65.32 (− 71.37, − 57.99)	− 60.38 (− 67.07, − 52.32)
Placebo-adjusted change (95% CI)	13.17 (− 12.89, 47.04)	− 1.77 (− 24.39, 27.63)	− 12.47 (− 32.87, 14.12)	–
*P* value^b^	0.3532	0.8935	0.3241	–

*CI* confidence interval, *FAS* full analysis set

^a^Differences are based on a mixed-effects model for the log-transformed ratio of the number of coughs/h at 24 h at each Visit with treatment, week, and treatment by week as fixed effects; patient as random effect; and region and the log-transformed coughs/h in 24 h at baseline as covariates. Modeled estimates are presented as percent change from baseline. Placebo-adjusted change is the percent change relative to placebo

^b^*P* value is based on the model described and was evaluated at the 2-sided alpha level of 0.05

In the PPS, placebo-adjusted changes from baseline in hourly cough counts in 24 h following 4 weeks of treatment were 10.66%, − 6.26%, and − 19.29% with sivopixant 50, 150, and 300 mg, respectively (*P* > 0.05) (e-Table 1, Online Resource). Percent changes in awake/asleep hourly cough counts from baseline to Week 4 were consistent with the 24-h data (e-Table 2; e-Table 3; Online Resource).

### Patient-Reported Outcomes

**Fig. 3:**
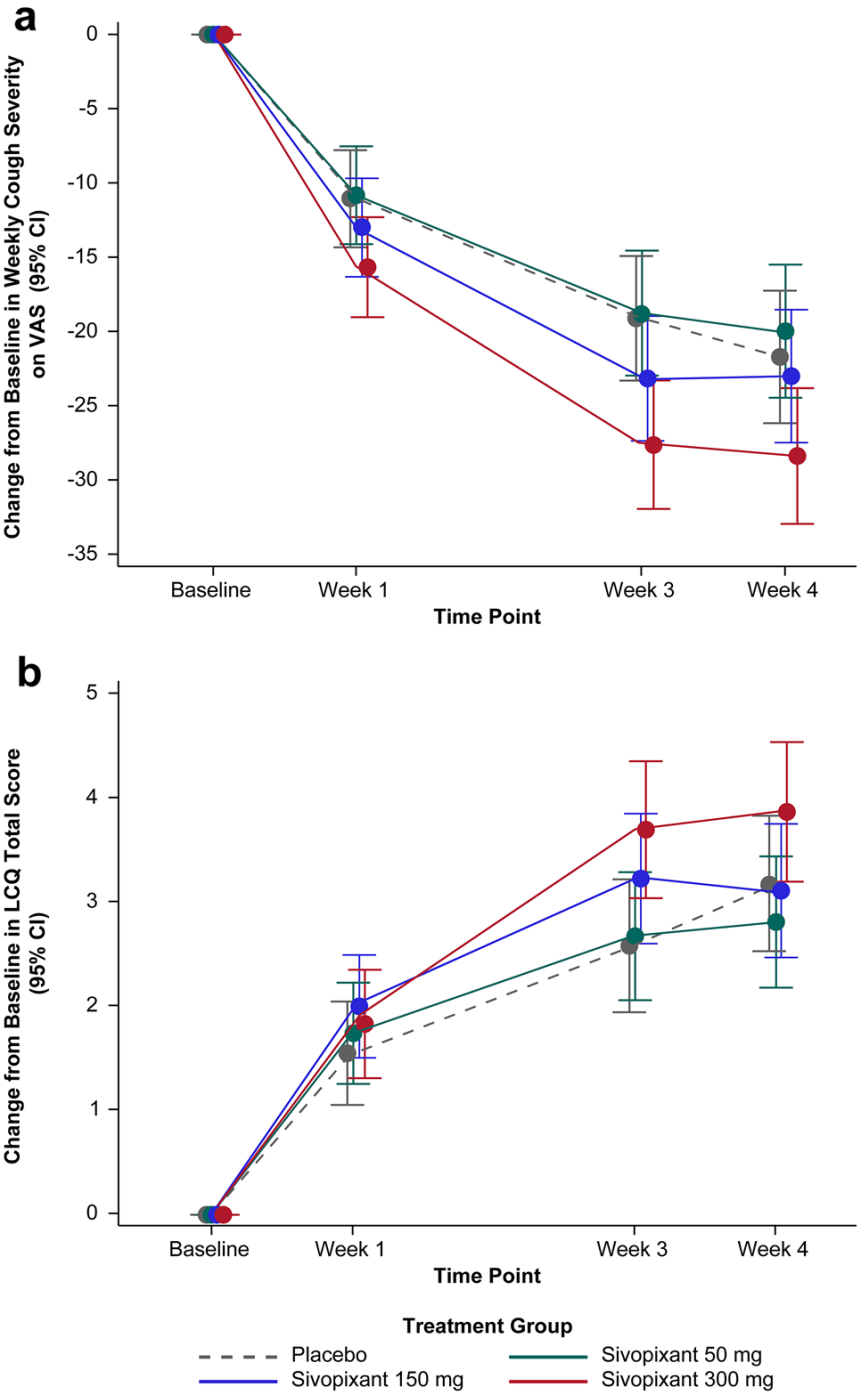
Change from baseline in **a** weekly cough severity (VAS) and **b** LCQ total score (FAS). Change from baseline in **a** weekly cough severity (VAS) and **b** LCQ total score (FAS). Treatment effects and their 95% CIs plotted are based on a mixed-effects model for the change from baseline in **a** VAS and **b** LCQ total score at Weeks 1, 3 and 4, with treatment group, week, and interaction between treatment group and week as fixed effects; patient as random effect; and region and the weekly cough severity on VAS/LCQ total score at baseline as covariates. Modeled estimates are presented as percent change from baseline. *CI* confidence interval, *FAS* full analysis set, *LCQ* Leicester Cough Questionnaire, *VAS* visual analog scale

Changes in cough severity (VAS) and LCQ total score were greatest with sivopixant 300 mg (Fig. 3). Placebo-adjusted changes in VAS at Week 4 were 1.75 mm (*P* = 0.5854), − 1.21 mm (*P* = 0.7056), and − 6.55 mm (*P* = 0.0433) with sivopixant 50, 150, and 300 mg, respectively (e-Table 4, Online Resource).

Placebo-adjusted changes from baseline to Week 4 in LCQ total score were − 0.37 (*P* = 0.4207), − 0.07 (*P* = 0.8806), and 0.69 (*P* = 0.1473) with sivopixant 50, 150, and 300 mg, respectively (e-Table 5, Online Resource). Overall, 61.6%, 68.3%, 76.9%, and 58.5% of patients receiving sivopixant 50, 150, and 300 mg and placebo, respectively, reported ≥ 1.3-point improvement (responders) in the LCQ total score at treatment completion. The percentage of responders in the sivopixant 300-mg group was 16.9% higher (risk difference, 16.9%; 95% CI 2.6, 31.2) than that in the placebo group (odds ratio [OR], 2.21; *P* = 0.0227) (Table 3).

**Table 3:** Improvement in LCQ and PGIC (FAS) from baseline following 4 weeks of study treatment

LCQ—≥ 1.3-point improvement in total score					
Response level treatment	Responders *n*/*N* (%)^a^	Risk differences^b^ % (95% CI)	Risk ratio^b^ (95% CI)	Odds ratio^b^ (95% CI)	*P* value^b^
Sivopixant 50 mg	53/86 (61.6)	2.1 (− 13.2, 17.4)	1.04 (0.81, 1.33)	1.09 (0.59, 1.99)	0.7824
Sivopixant 150 mg	56/82 (68.3)	9.5 (− 5.2, 24.3)	1.16 (0.92, 1.47)	1.51 (0.80, 2.88)	0.2046
Sivopixant 300 mg	60/78 (76.9)	16.9 (2.6, 31.2)	1.28 (1.04, 1.59)	2.21 (1.11, 4.39)	0.0227
Placebo	48/82 (58.5)	–	–	–	–

PGIC—any improvements					
Response level treatment	Responders *n*/*N* (%)^a^	Risk differences^b^ % (95% CI)	Risk ratio^b^ (95% CI)	Odds ratio^b^ (95% CI)	*P* value^b^
Sivopixant 50 mg	57/93 (61.3)	− 11.1 (− 24.9, 2.8)	0.85 (0.68, 1.05)	0.62 (0.34, 1.14)	0.1125
Sivopixant 150 mg	72/92 (78.3)	6.6 (− 5.9, 19.2)	1.09 (0.92, 1.29)	1.43 (0.73, 2.80)	0.3026
Sivopixant 300 mg	79/91 (86.8)	14.2 (2.7, 25.6)	1.20 (1.03, 1.39)	2.54 (1.17, 5.51)	0.0182
Placebo	65/91 (71.4)	–	–	–	–

*CI* confidence interval, *FAS* full analysis set, *LCQ* Leicester Cough Questionnaire, *PGIC* Patient Global Impression of Change

^a^LCQ: Patients were considered responders if they met the improvement from the specified baseline threshold (≥ 1.3-points). For PGIC: Patients were considered responders if they reported “Very much improved,” “Much improved,” or “Minimally improved” on the PGIC assessment

^b^Based on the Cochran-Mantel–Haenszel test stratified by region (Europe, the United States, or Japan) and cough count at baseline (≥ 30 coughs/h, < 30 coughs/h) vs placebo

Additionally, 61.3%, 78.3%, 86.8%, and 71.4% of patients receiving sivopixant 50, 150, 300 mg, and placebo, respectively, reported any improvements in PGIC. The percentage of patients who reported any improvement was 14.2% higher with sivopixant 300 mg (risk difference, 14.2%; 95% CI 2.7, 25.6) than with placebo (OR 2.54; *P* = 0.0182) (Table 3).

### Post Hoc Subgroup Analysis

**Fig. 4:**
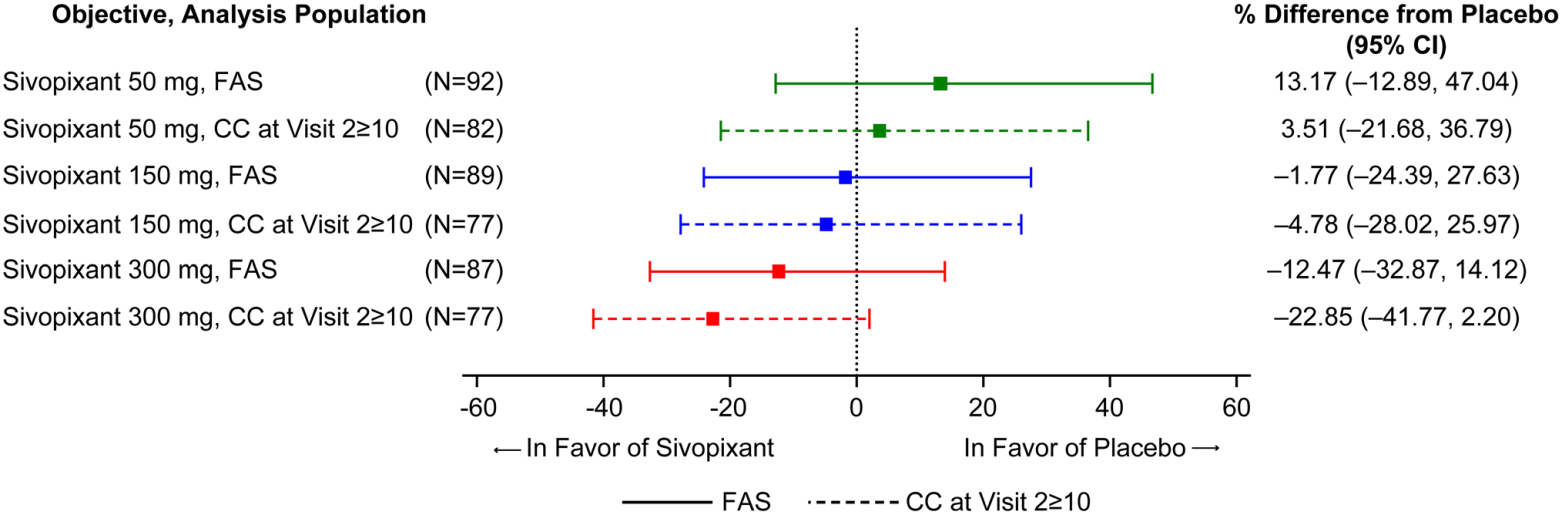
Percent change relative to placebo in hourly cough count in 24 h at Week 4: patients with a cough count of ≥ 10 at Visit 2 in the FAS. Treatment effects and their 95% CIs plotted are based on a mixed-effects model for the log-transformed ratio of the number of coughs/h in 24 h after 4 weeks of treatment, with treatment, week, and treatment by week as fixed effects; patient as random effect; and region and the log-transformed coughs/h in 24 h at baseline as covariates. Modeled estimates are presented as percent change relative to placebo from Visit 2. *CC* cough count, *CI* confidence interval, *FAS* full analysis set

In the subgroup of patients with ≥ 10 coughs/h at both screening Visits 1 and 2, placebo-adjusted changes in hourly cough counts in 24 h at Week 4 were 3.51%, − 4.78%, and − 22.85% with sivopixant 50, 150, and 300 mg, respectively (Fig. 4; e-Table 6, Online Resource).

In the small subgroup of patients with a cough frequency ≥ 10 coughs/h at screening Visit 1 that decreased to < 10 coughs/h at screening Visit 2 (sivopixant: 50 mg: 13 patients; 150 mg: 13 patients; 300 mg: 10 patients and placebo: 11 patients), no sivopixant arm showed improvements in cough reduction vs placebo (e-Table 7, Online Resource).

Similarly, consistently greater improvements were observed in VAS, LCQ total score, and PGIC with sivopixant vs placebo in patients with ≥ 10 coughs/h at screening Visits 1 and 2 than in patients with < 10 coughs/h at screening Visit 2 (e-Tables 8–11, Online Resource). In patients with ≥ 10 coughs/h at screening Visits 1 and 2, the percentage of patients who reported ≥ 1.3-point improvement in LCQ total score was 20.7% higher with sivopixant 300 mg than with placebo (OR 2.82; 95% CI 1.31, 6.07), and the percentage of patients who reported any improvements in PGIC was 18.7% higher with sivopixant 300 mg than with placebo (OR 3.59; 95% CI 1.51, 8.53) (e-Table 10, Online Resource). No improvements for any doses were observed for patients with < 10 coughs/h (e-Table 11, Online Resource).

### Safety

TEAEs were reported in 25.7%, 32.0%, 49.0%, and 20.6% of patients receiving sivopixant 50, 150, 300 mg, and placebo, respectively. No deaths or serious TEAEs were observed. All TEAEs experienced by patients receiving sivopixant were mild to moderate in severity. Study discontinuation due to TEAEs was low and reported in 8 patients (sivopixant 50 mg, *n* = 1 [1%]; sivopixant 150 mg, *n* = 1 [1%]; sivopixant 300 mg, *n* = 4 [4%]; and placebo, *n* = 2 [2%]). Three patients (3%) receiving sivopixant 300 mg discontinued treatment due to TEAEs of skin and subcutaneous tissue disorders. The most frequent TEAEs (≥ 5%) in any treatment group were taste-related TEAEs of dysgeusia and hypogeusia with sivopixant and headache with placebo. The incidence of taste-related TEAEs was similar between patients receiving sivopixant 50 mg (2.0%) and those receiving placebo (2.9%), and higher in patients receiving sivopixant 150 mg (13.6%) and 300 mg (33.0%). One patient receiving sivopixant 300 mg (1%) discontinued the study due to ageusia. Most taste-related TEAEs were mild in severity and occurred within 1 week of the study intervention, and most participants recovered during the study treatment or within 1 week of study treatment completion. All treatment-related TEAEs of taste-related events were reversible (Table 4).

**Table 4:** Safety outcomes

Number of patients, *n* (%)	Sivopixant			Placebo
	50 mg *N* = 101	150 mg *N* = 103	300 mg *N* = 100	*N* = 102
Any TEAE leading to treatment discontinuation	1 (1.0)	1 (1.0)	4 (4.0)	2 (2.0)
Any TEAE	26 (25.7)	33 (32.0)	49 (49.0)	21 (20.6)
At least 1 taste-related TEAE^a^	2 (2.0)	14 (13.6)	33 (33.0)	3 (2.9)
Dysgeusia	2 (2.0)	12 (11.7)	27 (27.0)	3 (2.9)
Hypogeusia	0	1 (1.0)	6 (6.0)	0
Taste disorder	0	1 (1.0)	1 (1.0)	0
Ageusia	0	0	1 (1.0)	0

*TEAE* treatment-emergent adverse event

^a^Severity of taste-related adverse events: All events were mild except the following number of cases of moderate dysgeusia: sivopixant 150 mg, *n* = 2 (1.9%); sivopixant 300 mg, *n* = 3 (3.0%); placebo, *n* = 1 (1.0%)

## Discussion

This multinational, multicenter, randomized, controlled phase 2b trial assessed the impact of different doses of the P2X3 receptor antagonist sivopixant on patients with RCC/UCC over a 4-week period. No sivopixant dose showed a statistically significant difference vs placebo for 24-h cough count at the end of 4 weeks, and the primary study endpoint was not met. Nonetheless, sivopixant showed a dose-dependent effect in reducing the 24-h cough frequency. After 4 weeks of treatment, sivopixant 300 mg showed greater reductions in hourly cough counts over 24 h than placebo, with a − 12.47% placebo-adjusted change. Patient-reported outcomes (cough severity [VAS], LCQ total score, and PGIC responder analyses) suggest that sivopixant 300 mg may be effective for RCC/UCC. Along with the well-tolerated safety profile, sivopixant 300 mg shows promising potential for evaluation in further clinical trials.

While a response to placebo has been seen frequently in other CC trials, improvement in the 24-h cough count (at Week 4) in the placebo group in this study was larger than that reported in other CC trials, with a − 60.38% improvement in 24-h cough count vs a − 31.4% improvement at Week 2 in the phase 2a study [18]. The reasons for higher placebo response are unclear but could be related to the patient population and expectation bias. Patients who experienced spontaneous improvement or improved adherence to background medication during the clinical trial may have been included, further contributing to the high placebo response.

The observed absolute reductions in cough frequency with sivopixant in this study are consistent with other P2X3 receptor antagonist studies [26–28]. However, placebo-adjusted differences are smaller, driven by the larger reduction in cough frequency observed in the placebo-treated arm. Gefapixant 45 mg twice daily resulted in a 24-h placebo-adjusted change of − 18.5% at Week 12 in the COUGH-1 trial and − 14.6% at Week 24 in the COUGH-2 trial [28]. In other studies, larger treatment effects have been found with smaller placebo effects [26, 27].

A large proportion of subjects in the study were on concomitant medications to treat conditions with a potential effect on cough (approximately 70% across all treatment arms). Better compliance with these medications might have contributed to the lack of separation between active treatment and placebo. In a post hoc analysis (data not shown), the relative change in 24-h cough count vs placebo was very small in the subgroup with concomitant medications and was numerically larger in favor of sivopixant in the subgroup without concomitant medications. However, contrary to this notion, the magnitude of the improvement on placebo was similar in both subgroups. Careful monitoring of the treatments for comorbidities affecting cough and the use of concomitant medications might be advisable for future studies in RCC/UCC.

The phase 2a study identified that patients with < 10 coughs/h at baseline showed highly variable values of cough count during the trial, although the patient number was small. In this phase 2b study, the study protocol required a minimum of 10 coughs/h at the beginning of the screening period (Visit 1) but not at Visit 2, which was close to randomization and regarded as baseline. The 24-h cough count data at Visit 2 were not available by the time of randomization to exclude those with a cough frequency of < 10 coughs/h. Therefore, we conducted a post hoc analysis to exclude patients with < 10 coughs/h at Visit 2. The subgroup of patients with a 24-h cough frequency of ≥ 10 coughs/h at both screening Visits 1 and 2 had greater improvements in cough with sivopixant 300 mg at Week 4 achieving a − 22.85% placebo-adjusted change in hourly cough counts over 24 h. Based on this finding, we considered that a baseline cough count of ≥ 10 coughs/h might be necessary to detect a treatment response to sivopixant. In addition, other CC trials reported that baseline cough counts were related to efficacy [29, 30]. However, our additional subgroup analyses conducted at a higher cutoff value for 24-h cough frequency at Visit 2 (< or ≥ 15, 20, 25, 35, or 40 coughs/h) showed no clear trend between the subgroups in treatment efficacy (data not shown). Therefore, we cannot conclude that sivopixant has higher efficacy in patients with a higher baseline cough count.

Changes in cough severity (VAS), LCQ total score, and PGIC were more likely to show improvements with sivopixant 300 mg vs placebo. A significantly higher proportion of patients receiving sivopixant 300 mg achieved the minimal clinically important difference of a ≥ 1.3-point improvement [25, 31] in LCQ (OR 2.21; 95% CI 1.11, 4.39; *P* = 0.0227) or any improvements in PGIC (OR 2.54; 95% CI 1.17, 5.51; *P* = 0.0182) vs placebo after 4 weeks of treatment. The difference in the VAS, although statistically significant in favor of 300 mg vs placebo, was numerically small and of questionable clinical significance.

Taste-related TEAEs are expected with P2X3 receptor antagonists [28, 32]. In the current study, sivopixant was generally well tolerated, with 33.0% of patients reporting taste-related TEAEs with sivopixant 300 mg. Taste-related TEAEs increased in a dose-dependent manner; however, most of the events were mild, with few being moderate, and only one patient in the sivopixant 300 mg group discontinued treatment due to ageusia. Specific blockade of P2X3 receptors is not expected to result in a marked impairment in taste perception [18]. In this study, taste-related TEAEs with sivopixant 300 mg were lower [28] or comparable to outcomes in other P2X3 receptor antagonist trials [26, 27, 32, 33]. Studies with treatment duration > 4 weeks are needed to further examine taste-related TEAEs associated with sivopixant.

A strength of this study is the relatively large sample size of a broad population of patients with RCC/UCC from diverse geographical backgrounds, which supports data generalizability. This may mean that more patients were naïve to antitussive therapy. However, high placebo responses suggest that some patients might have had clinical courses not typical of RCC/UCC, and a high expectation bias may have played a role. Study limitations include a high placebo response and uncertainty regarding the clinical significance of the differences seen in the VAS assessment. Furthermore, objective measurement of cough frequency may not capture other features of cough such as cough intensity, which can only be assessed subjectively.

## Conclusions

Sivopixant did not demonstrate a statistically significant difference vs placebo in change from baseline in 24-h cough frequency. Sivopixant 300 mg was well tolerated and demonstrated greater reductions in hourly cough counts over 24 h than lower doses tested or placebo, although no significant differences were observed across groups. Therefore, efficacy has not been confirmed. Cough frequency at baseline (< or ≥ 10 coughs/h) and its consistency from the screening period may be factors impacting the ability to show treatment efficacy. Cough severity (VAS), LCQ total score, and PGIC showed significant improvement in responses with sivopixant 300 mg vs placebo. A well-tolerated safety profile with mild-to-moderate reversible taste disturbances was reported. This study lays the foundation for further evaluation of sivopixant 300 mg in future RCTs, with protocol planning informed by this phase 2b study, including measures to reduce the placebo effect.

## Supplementary Information

Below is the link to the electronic supplementary material.

Supplementary file1 (TIF 2330 KB)

Supplementary file2 (DOCX 178 KB)

## Data Availability

The data that support the findings of this study are available from the corresponding author upon reasonable request.
