# Supplementary figures and images for: A Randomized, Double-Blind, Placebo-Controlled, Parallel-Group Phase 2b Trial of P2X3 Receptor Antagonist Sivopixant for Refractory or Unexplained Chronic Cough
Source: Lung. 2022 Dec 13;201(1):25–35. doi: 10.1007/s00408-022-00592-5 (PMC9745691; doi:10.1007/s00408-022-00592-5)

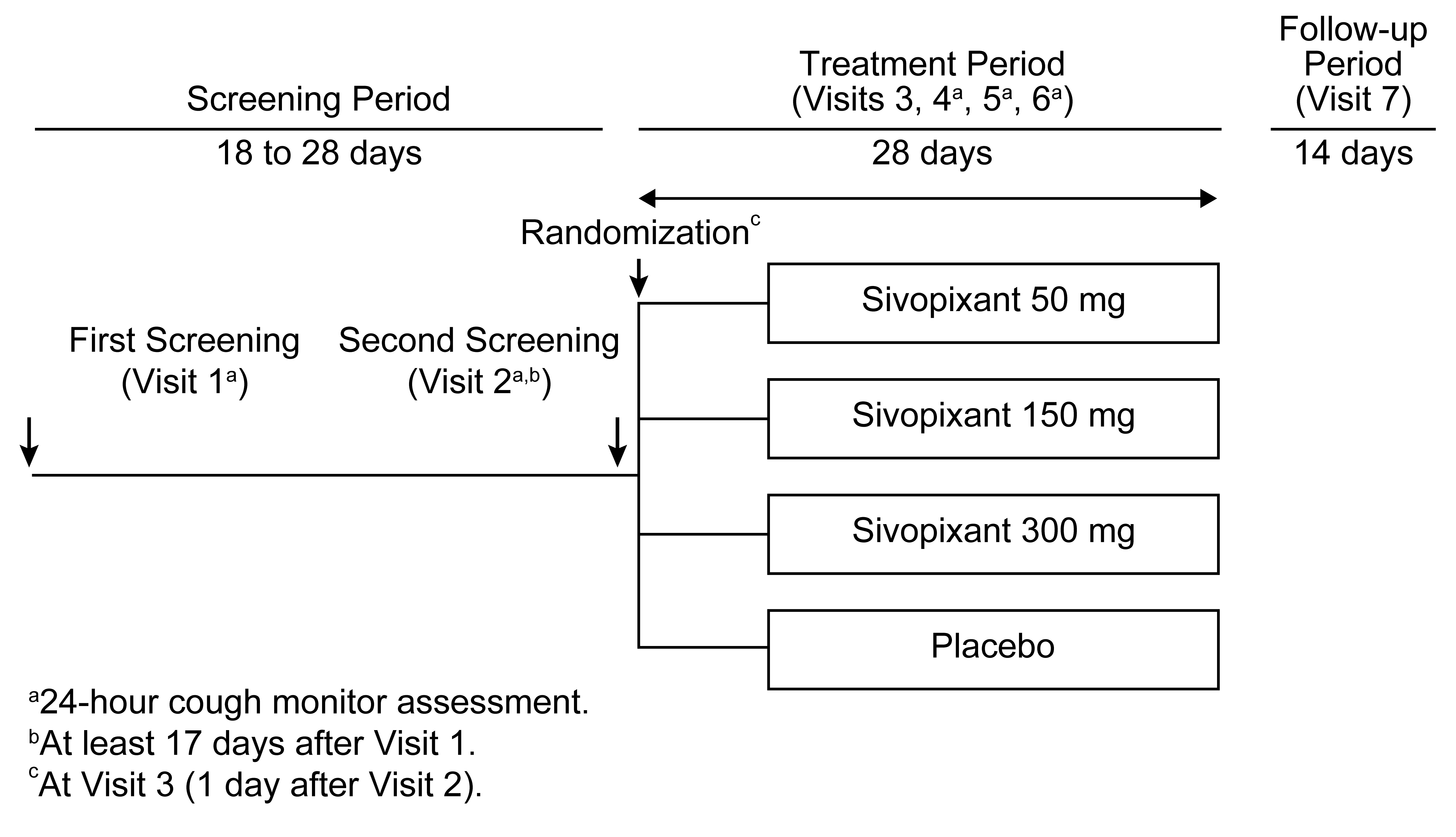

Supplement: Supplementary file 1 — Supplementary file1 (TIF 2330 KB) [file 408_2022_592_MOESM1_ESM.tif]
