# Supplementary material for: A Randomized, Double-Blind, Placebo-Controlled, Parallel-Group Phase 2b Trial of P2X3 Receptor Antagonist Sivopixant for Refractory or Unexplained Chronic Cough
Source: Lung. 2022 Dec 13;201(1):25–35. doi: 10.1007/s00408-022-00592-5 (PMC9745691; doi:10.1007/s00408-022-00592-5)
Supplement: Supplementary file 2 — Supplementary file2 (DOCX 178 KB) [file 408_2022_592_MOESM2_ESM.docx]

**A Randomized, Double-blind, Placebo-controlled, Parallel**-**group Phase 2b Trial of P2X3 Receptor Antagonist Sivopixant for Refractory or Unexplained Chronic Cough**

## Journal name: *Lung*

Lorcan McGarvey^1^, Jaclyn A. Smith^2^, Alyn Morice^3^, Surinder S. Birring^4^, Kian Fan Chung^5^, Peter V. Dicpinigaitis^6^, Akio Niimi^7^, Michael S. Benninger^8^, Mandel Sher^9^, Yuko Matsunaga^10^, Sayaka Miyazaki^11^, Mitsuaki Machida^11^, Hiroyuki Ishihara^11^, Adnan Mahmood^12^, Juan-Carlos Gomez^12^

**Affiliations**

^1^Queen’s University Belfast, Belfast, Northern Ireland, UK; ^2^University of Manchester and Manchester University NHS Foundation Trust, Manchester, UK; ^3^University of Hull Castle Hill Hospital, Hull, UK; ^4^King’s College Hospital, London, UK; ^5^National Heart & Lung Institute, Imperial College London & Royal Brompton and Harefield Hospitals, London, UK; ^6^Albert Einstein College of Medicine; Montefiore Medical Center, Division of Critical Care Medicine, Bronx, New York, USA; ^7^Department of Respiratory Medicine, Allergy and Clinical Immunology, Nagoya City University, Nagoya, Japan; ^8^Head and Neck Institute, The Cleveland Clinic, Cleveland, Ohio, USA; ^9^University of South Florida, Tampa, Florida, USA; ^10^Shionogi Inc., Florham Park, New Jersey, USA; ^11^Shionogi & Co., Ltd., Osaka, Japan; ^12^Shionogi B.V., London, UK.

Yuko Matsunaga is a former employee of Shionogi Inc.

**Corresponding author**

**Name:** Juan-Carlos Gomez

**Full mailing address**: Shionogi Europe, 33 Kingsway, London WC2B 6UF, UK

**Email address:** juan.carlos.gomez@shionogi.eu

## e-Figure 1 Study design

## Patients self-administered their first dose of the study drug after randomization. The study duration for each patient was approximately 8–10 weeks (screening, 18–28 days; treatment, 28 days; follow up, 14 days)


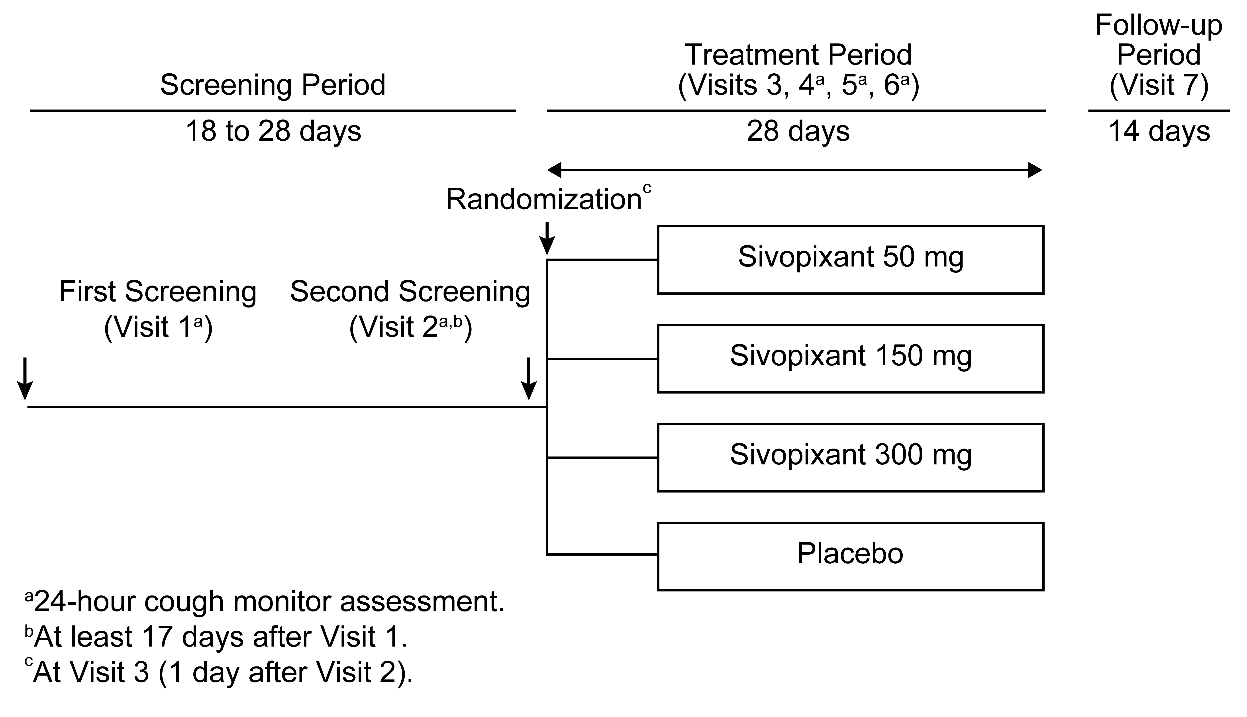


####

## e-Appendix 1 Inclusion/Exclusion Criteria

**Inclusion Criteria**

- Willing to comply with all study procedures and capable of giving signed informed consent.
- Male or female outpatient ≥18 to ≤80 years of age at the time of signing the informed consent form.
- Refractory chronic cough lasting for at least 1 year prior to Visit 1, defined as insufficient improvement in cough after treatment for the underlying condition(s) causing the cough or unexplained cough for which an underlying condition had not been determined.
- Severity of cough assessed as ≥40 mm on the visual analog scale at both Visit 1 and Visit 2.
- Cough count ≥10 times/h based on the 24-h cough count recording at Visit 1.

**Exclusion Criteria**

- Missing an entry in the patient electronic diary for the number of coughs on more than 30% of the days from the day of Visit 1 up to the day of Visit 2.
- Failure to obtain the cough count recording on the cough monitor at Visit 1 or Visit 2 for any reason, including device malfunction, based on initial assessment at the study site.
- Currently smoking (including, but not limited to, e-cigarettes, smokeless cigarettes, and vaping) or had used any inhalational agents (including, but not limited to, marijuana) that were potential irritants or had smoked or used any inhalational agents that were potential irritants in the 1 year prior to Visit 1 or had a smoking history of ≥20 pack‑years.
- Produced a significant amount of sputum suggestive of infection, bronchiectasis, chronic bronchitis, and so on.
- In the 4 weeks prior to Visit 1 or during screening, had a history of infection in the upper or lower respiratory tract, a significant change in lung function, or a pulmonary condition based on the judgment of the investigator.
- Chronic obstructive pulmonary disease or, as defined in the Global Initiative for Asthma 2019, had uncontrolled asthma symptoms (excluding cough).
- A clinically unstable medical condition or any other medical condition that in the opinion of the investigator would interfere with study participation or assessment of efficacy and safety.
- A ratio of forced expiratory volume in 1 s/forced vital capacity <60% at Visit 1.
- Any finding on chest x-ray or chest computed tomography scan (performed not more than 1 year prior to Visit 1 after the onset of chronic cough at Visit 1) that could be considered the cause of chronic cough or indicative of lung disease.

## e-Appendix 2 Efficacy Assessment

Efficacy was assessed based on the following:

1. The objective measurement of cough counts using a cough monitor.
2. Questionnaires or instruments routinely used in studies in this patient population. The patient was to complete these questionnaires and instruments in an electronic tablet and in an electronic diary.

Number of Coughs (VitaloJAK™ Cough Monitor)

The number of coughs over a 24-h period was recorded at Visits 1, 2, 4, 5, and 6 using the VitaloJAK™ cough monitor [1]. The cough monitor was applied to the patient at each of these visits, and the patient was instructed to remove the monitor after 24 h. The number of coughs was measured (including a classification of awake and asleep hours) by cough analysis technicians for each of the following periods: 24 h, waking hours, and sleeping hours.

Cough Severity as Assessed on the Visual Analog Scale (VAS)

Cough VAS (0–100 mm; 0: no cough, 100: worst cough ever) was used to assess cough severity [2]. Weekly severity of cough (electronic tablet): The patient was asked to provide his/her assessment of the severity of cough during the past week at Visits 1, 2, 4, 5, and 6 and/or, if applicable, at the time of discontinuation of the study treatment on the VAS.

Leicester Cough Questionnaire (LCQ; Electronic Tablet)

The LCQ is a patient-reported quality-of-life measure of chronic cough [3]. The questionnaire

consists of 19 items to which the patient responds on a 7-point Likert response scale. The patient was asked to complete the LCQ at Visits 1, 2, 4, 5, and 6 and/or, if applicable, at the time of discontinuation of the study drug.

Patient Global Impression of Change (PGIC; Electronic Tablet)

The PGIC is a patient-reported measure of overall health status and consists of 1 item adapted from the Clinical Global Impressions scale [4]. The patient was asked to complete the PGIC at Visit 6 or, if applicable, at the early discontinuation visit.

**References**

1. Smith JA, Holt K, Dockry R, et al (2021) Performance of a digital signal processing algorithm for the accurate quantification of cough frequency. Eur Respir J 58:2004271. <https://doi.org/10.1183/13993003.04271-2020>
2. Morice AH, Fontana GA, Belvisi MG, et al (2007) ERS guidelines on the assessment of cough. Eur Respir J 29:1256–1276. <https://doi.org/10.1183/09031936.00101006>
3. Birring SS, Prudon B, Carr AJ, Singh SJ, Morgan MD, Pavord ID (2003) Development of a symptom specific health status measure for patients with chronic cough: Leicester Cough Questionnaire (LCQ). Thorax 58:339–343. <http://doi.org/10.1136/thorax.58.4.339>
4. Martin Nguyen A, Bacci E, Dicpinigaitis P, Vernon M (2020) Quantitative measurement properties and score interpretation of the cough severity diary in patients with chronic cough. Ther Adv Respir Dis 14:1753466620915155. <https://doi.org/10.1177/1753466620915155>

**e-Appendix 3 Description of Analysis Sets**

Full analysis set (FAS): Included all randomized patients who received ≥1 dose of the study medication and had a valid cough monitor assessment at both baseline and ≥1 visit after the study treatment initiation.

Per-protocol set (PPS): Included all randomized patients in the FAS who fulfilled all the eligibility criteria with no major protocol deviations or use of prohibited therapy that could affect evaluation of efficacy.

Safety population: Included all randomized patients who received ≥1 dose of the study medication.

## e-Table 1 Percent change in hourly cough counts in 24 h from baseline at week 1, week 3, and week 4 (PPS)

| Modeled estimates^a^ | Sivopixant | | | Placebo |
| --- | --- | --- | --- | --- |
|  | 50 mg  N=92 | 150 mg  N=92 | 300 mg  N=85 | N=95 |
| Week 1 | n=91 | n=86 | n=81 | n=94 |
| Change, % (95% CI) | −37.09 (−45.61, −27.23) | −46.06 (−53.44, −37.52) | −51.49 (−58.33, −43.52) | −47.29 (−54.33, −39.17) |
| Placebo-adjusted change (95% CI) | 19.36 (−2.51, 46.13) | 2.33 (−16.53, 25.45) | −7.96 (−25.23, 13.29) | - |
| *P* value^b^ | 0.0863 | 0.8243 | 0.4327 | - |
| Week 3 | n=85 | n=86 | n=81 | n=89 |
| Change, % (95% CI) | −52.58 (−60. 77, −42. 69) | −57.77 (−65.05, −48.97) | −64.63 (−70.93, −56.98) | −51.66 (−59.88, −41.75) |
| Placebo-adjusted change (95% CI) | −1.92 (−24.71, 27.76) | −12.65 (−32.94, 13.79) | −26.84 (−44.13, −4.21) | - |
| *P* value^b^ | 0.8854 | 0.3153 | 0.0232 | - |
| Week 4 | n=86 | n=82 | n=80 | n=88 |
| Change, % (95% CI) | −55.03 (−63.00, −45.35) | −61.90 (−68.69, −53.65) | −67.20 (−73.21, −59.85) | −59.36 (−66.47, −50.74) |
| Placebo-adjusted change (95% CI) | 10.66 (−15.73, 45.30) | −6.26 (−28.69, 23.23) | −19.29 (−38.89, 6.58) | - |
| *P* value^b^ | 0.4652 | 0.6424 | 0.1304 | - |

^a^Differences are based on a mixed model for the log-transformed ratio of the number of coughs/h in 24 h at each visit, with treatment, week, and treatment by week as fixed effects; patient as random effect; and region (Europe, the United States, or Japan) and the log-transformed coughs/h in 24 h at baseline as covariates. Modeled estimates are presented as percent change from baseline. Placebo-adjusted change is the percent change relative to placebo. ^b^*P* value is based on the model described and is evaluated at the 2-sided alpha level of 0.05. *CI* confidence interval, *PPS* per-protocol set.

## e-Table 2 Percent change in hourly cough counts during awake hours at week 1, week 3, and week 4 (FAS)

| Modeled estimates^a^ | Sivopixant | | | Placebo |
| --- | --- | --- | --- | --- |
|  | 50 mg  N=100 | 150 mg  N=102 | 300 mg  N=96 | N=102 |
| Baseline | n=100 | n=102 | n=96 | n=102 |
|  |  |  |  |  |
| Geometric mean (95% CI) | 33.90 (29.65, 38.75) | 34.18 (28.19, 41.45) | 35.85 (30.59, 42.01) | 34.46 (29.44, 40.34) |
| Week 1 | n=98 | n=94 | n=91 | n=101 |
| Change, % (95% CI) | −37.41 (−45.41, −28.24) | −45.98 (−52.90, −38.03) | −49.66 (−56.26, −42.07) | −46.34 (−53.11, −38.59) |
| Placebo-adjusted change (95% CI) | 16.65 (−3.57, 41.11) | 0.68 (−16.82, 21.87) | −6.19 (−22.67, 13.81) | - |
| *P* value^b^ | 0.1125 | 0.9442 | 0.5162 | - |
| Week 3 | n=93 | n=94 | n=90 | n=96 |
| Change, % (95% CI) | −52.42 (−60.58, −42.57) | −58.30 (−65.41, −49.72) | −63.21 (−69.65, −55.41) | −54.28 (−62.05, −44.92) |
| Placebo-adjusted change (95% CI) | 4.07 (−20.04, 35.45) | −8.78 (−29.86, 18.64) | −19.53 (−38.37, 5.05) | - |
| *P* value^b^ | 0.7660 | 0.4923 | 0.1098 | - |
| Week 4 | n=92 | n=89 | n=87 | n=95 |
| Change, % (95% CI) | −55.77 (−63.61, −46.25) | −61.75 (−68.52, −53.52) | −66.33 (−72.43, −58.89) | −60.31 (−67.27, −51.87) |
| Placebo-adjusted change (95% CI) | 11.44 (−15.19, 46.42) | −3.62 (−26.64, 26.63) | −15.17 (−35.67, 11.86) | - |
| *P* value^b^ | 0.4359 | 0.7907 | 0.2429 | - |

^a^Differences are based on a mixed model for the log-transformed ratio of the number of coughs/h while awake with treatment, week, and treatment by week as fixed effects; patient as random effects; and region (Europe, the United States, or Japan) and the log-transformed coughs/h while awake at baseline as covariates. Modeled estimates are presented as percent change from baseline. Placebo-adjusted change is the percent change relative to placebo. ^b^*P* value is based on the model described and was evaluated at the 2-sided alpha level of 0.05. *CI* confidence interval, *FAS* full analysis set.

## e-Table 3 Percent change in hourly cough counts during asleep hours at week 1, week 3, and week 4 (FAS)

| Modeled estimates^a^ | Sivopixant | | | Placebo |
| --- | --- | --- | --- | --- |
|  | 50 mg  N=100 | 150 mg  N=102 | 300 mg  N=96 | N=102 |
| Baseline | n=100 | n=102 | n=95 | n=102 |
|  |  |  |  |  |
| Geometric mean (95% CI) | 3.41 (2.63, 4.43) | 2.84 (2.10, 3.84) | 2.70 (2.05, 3.54) | 2.98 (2.30, 3.85) |
| Week 1 | n=97 | n=93 | n=87 | n=99 |
| Change, % (95% CI) | −36.09 (−51.38, −16.00) | −42.81 (−56.65, −24.54) | −56.62 (−67.42, −42.24) | −47.10 (−59.64, −30.67) |
| Placebo-adjusted change (95% CI) | 20.81 (−17.58, 77.09) | 8.12 (−26.46, 58.96) | −17.99 (−44.59, 21.38) | - |
| *P* value^b^ | 0.3315 | 0.6905 | 0.3206 | - |
| Week 3 | n=93 | n=93 | n=88 | n=95 |
| Change, % (95% CI) | −57.58 (−68.07, −43.66) | −57.56 (−68.05, −43.63) | −53.91 (−65.58, −38.28) | −57.86 (−68.18, −44.18) |
| Placebo-adjusted change (95% CI) | 0.65 (−32.36, 49.78) | 0.70 (−32.32, 49.84) | 9.37 (−26.94, 63.72) | - |
| *P* value^b^ | 0.9745 | 0.9725 | 0.6626 | - |
| Week 4 | n=92 | n=87 | n=86 | n=93 |
| Change, % (95% CI) | −54.43 (−66.61, −37.80) | −53.17 (−65.89, −35.73) | −65.48 (−74.96, −52.41) | −52.73 (−65.30, −35.61) |
| Placebo-adjusted change (95% CI) | −3.60 (−37.70, 49.17) | −0.94 (−36.25, 53.92) | −26.97 (−53.15, 13.84) | - |
| *P* value^b^ | 0.8690 | 0.9664 | 0.1646 | - |

^a^Differences are based on a mixed model for the log-transformed ratio of the number of coughs/h while asleep with treatment, week, and treatment by week as fixed effects; patient as random effect; and region (Europe, the United States, or Japan) and the log-transformed coughs/h while asleep at baseline as covariates. Modeled estimates are presented as percent change from baseline. Placebo-adjusted change is the percent change relative to placebo. ^b^*P* value is based on the model described and was evaluated at the 2-sided alpha level of 0.05. *CI* confidence interval, *FAS* full analysis set

## e-Table 4 Change from baseline in weekly cough severity (VAS) at week 1, week 3, and week 4 (FAS)

| Modeled estimates^a^ | Sivopixant | | | Placebo |
| --- | --- | --- | --- | --- |
|  | 50 mg  N=100 | 150 mg  N=102 | 300 mg  N=96 | N=102 |
| Week 1 | n=96 | n=95 | n=91 | n=101 |
| Change, (95% CI) | −10.83 (−14.13, −7.54) | −12.98 (−16.27, −9.69) | −15.64 (−19.01, −12.27) | −11.02 (−14.26, −7.78) |
| Placebo-adjusted change (95% CI) | 0.18 (−4.40, 4.77) | −1.96 (−6.54, 2.62) | −4.62 (−9.25, 0.01) | - |
| *P* value^b^ | 0.9369 | 0.4003 | 0.0503 | - |
| Week 3 | n=97 | n=95 | n=91 | n=95 |
| Change, (95% CI) | −18.74 (−22.94, −14.53) | −23.09 (−27.29, −18.89) | −27.55 (−31.86, −23.24) | −19.07 (−23.27, −14.88) |
| Placebo-adjusted change (95% CI) | 0.33 (-5.58, 6.25) | −4.02 (−9.93, 1.89) | −8.48 (−14.46, −2.50) | - |
| *P* value^b^ | 0.9115 | 0.1817 | 0.0056 | - |
| Week 4 | n=93 | n=92 | n=91 | n=93 |
| Change, (95% CI) | −19.99 (−24.47, −15.51) | −22.95 (−27.42, −18.47) | −28.29 (−32.86, −23.72) | −21.74 (−26.20, −17.27) |
| Placebo-adjusted change (95% CI) | 1.75 (−4.55, 8.05) | −1.21 (−7.50, 5.08) | −6.55 (−12.91, −0.20) | - |
| *P* value^b^ | 0.5854 | 0.7056 | 0.0433 | - |

^a^Differences are based on a mixed model for the change in weekly cough severity score after 4 weeks of treatment with treatment, week, and treatment-by-week as fixed effects; patient as random effect; and region (Europe, the United States, or Japan) and the severity score at baseline as covariates. ^b^*P* value is based on the model described and was evaluated at the 2-sided alpha level of 0.05. *CI* confidence interval, *FAS* full analysis set, *VAS* visual analog scale

## e-Table 5 Change from baseline in LCQ total score at week 1, week 3, and week 4 (FAS)

| Modeled estimates^a^ | Sivopixant | | | Placebo |
| --- | --- | --- | --- | --- |
|  | 50 mg  N=100 | 150 mg  N=102 | 300 mg  N=96 | N=102 |
| Week 1 | n=89 | n=86 | n=77 | n=87 |
| Change, (95% CI) | 1.74 (1.26, 2.23) | 2.00 (1.50, 2.49) | 1.83 (1.31, 2.35) | 1.55 (1.05, 2.04) |
| Placebo-adjusted change (95% CI) | 0.20 (−0.50, 0.89) | 0.45 (−0.25, 1.15) | 0.28 (−0.43, 1.00) | - |
| *P* value^b^ | 0.5775 | 0.2055 | 0.4399 | - |
| Week 3 | n=90 | n=87 | n=78 | n=82 |
| Change, (95% CI) | 2.67 (2.05, 3.28) | 3.22 (2.60, 3.85) | 3.69 (3.03, 4.35) | 2.58 (1.94, 3.22) |
| Placebo-adjusted change (95% CI) | 0.09 (−0.79, 0.97) | 0.65 (−0.25, 1.54) | 1.11 (0.20, 2.03) | - |
| *P* value^b^ | 0.8414 | 0.1552 | 0.0173 | - |
| Week 4 | n=86 | n=82 | n=78 | n=82 |
| Change, (95% CI) | 2.80 (2.17, 3.43) | 3.10 (2.46, 3.75) | 3.86 (3.19, 4.53) | 3.17 (2.52, 3.82) |
| Placebo-adjusted change (95% CI) | −0. 37 (−1.28, 0.53) | −0.07 (−0.98, 0.84) | 0.69 (−0.24, 1.62) | - |
| *P* value^b^ | 0.4207 | 0.8806 | 0.1473 | - |

^a^Differences are based on a mixed model for the change in LCQ total score after 4 weeks of treatment with treatment, week, and treatment-by-week as fixed effects; patient as random effect; and region (Europe, the United States, or Japan) and the LCQ score of corresponding domain at baseline as covariates. Modeled estimates are presented as percent improvement from baseline. Placebo-adjusted change is the improvement relative to placebo. ^b^*P* value is based on the model described and was evaluated at the 2-sided alpha level of 0.05. *CI* confidence interval, *FAS* full analysis set, *LCQ* Leicester Cough Questionnaire

## **e-Table 6** Percent change in hourly cough counts in 24 hours at week 1, week 3, and week 4 (subgroup analysis: hourly cough count ≥10 at Visit 2 in the FAS)

| Modeled estimates^a^ | Sivopixant | | | Placebo |
| --- | --- | --- | --- | --- |
|  | 50 mg | 150 mg | 300 mg |  |
| Week 1 | n=85 | n=81 | n=82 | n=91 |
| Change, % (95% CI) | −39.86 (−48.02, −30.41) | −45.88 (−53.27, −37.32) | −53.90 (−60.20, −46.60) | −46.28 (−53.40, −38.07) |
| Placebo-adjusted change (95% CI) | 11.95 (−8.50, 36.97) | 0.74 (−17.74, 23.37) | −14.19 (−29.95, 5.12) | - |
| Week 3 | n=82 | n=82 | n=80 | n=86 |
| Change, % (95% CI) | −54.56 (−62.51, −44.94) | −58.79 (−65.98, −50.08) | −65.97 (−71.97, −58.69) | −53.84 (−61.76, −44.27) |
| Placebo-adjusted change (95% CI) | −1.57 (−24.67, 28.61) | −10.73 (−31.67, 16.62) | −26.28 (−43.68, −3.52) | - |
| Week 4 | n=82 | n=77 | n=77 | n=84 |
| Change, % (95% CI) | −58.25 (−65.82, −49.02) | −61.60 (−68.60, −53.03) | −68.89 (−74.60, −61.89) | −59.67 (−66.86, −50.91) |
| Placebo-adjusted change (95% CI) | 3.51 (−21.68, 36.79) | −4.78 (−28.02, 25.97) | −22.85 (−41.77, 2.20) | - |

^a^Differences are based on a mixed model for the log-transformed ratio of the number of coughs/h in 24 h at each visit with treatment, week, and treatment by week as fixed effect; patient as random effects; and region (Europe, the United States, or Japan) and the log-transformed coughs/h in 24 h at baseline as covariates. Modeled estimates are presented as percent change from baseline. Placebo-adjusted change is the percent change relative to placebo. *CI* confidence interval, *FAS* full analysis set

## e-Table 7 Percent change in hourly cough counts in 24 hours at week 1, week 3, and week 4 (subgroup analysis: hourly cough count <10 at Visit 2 in the FAS)

| Modeled estimates^a^ | Sivopixant | | | Placebo |
| --- | --- | --- | --- | --- |
|  | 50 mg | 150 mg | 300 mg |  |
| Week 1 | n=13 | n=12 | n=9 | n=10 |
| Change, % (95% CI) | −14.16 (−43.01, 29.31) | −51.82 (-68.64, −25.99) | −3.98 (−41.78, 58.35) | −48.43 (−67.67, −17.76) |
| Placebo-adjusted change (95% CI) | 66.47 (−10.86, 210.87) | −6.57 (−50.05, 74.77) | 86.20 (−6.31, 270.06) | - |
| Week 3 | n=11 | n=12 | n=10 | n=10 |
| Change, % (95% CI) | −34.94 (−63.14, 14.82) | −51.49 (−71.90, −16.25) | −33.53 (−64.24, 23.55) | −53.85 (−74.43, −16.70) |
| Placebo-adjusted change (95% CI) | 40.96 (−37.89, 219.93) | 5.11 (−52.87, 134.42) | 44.03 (−38.71, 238.45) | - |
| Week 4 | n=10 | n=12 | n=10 | n=11 |
| Change, % (95% CI) | −24.14 (−53.56, 23.92) | −61.42 (−75.79, −38.53) | −15.14 (−50.08, 44.25) | −66.83 (−79.79, −45.56) |
| Placebo-adjusted change (95% CI) | 128.73 (13.88, 359.41) | 16.32 (−40.90, 128.96) | 155.87 (24.07, 427.70) | - |

^a^Differences are based on a mixed model for the log-transformed ratio of the number of coughs/h in 24-h at each visit with treatment, week, and treatment by week as fixed effects; patient as random effect; and region (Europe, the United States, or Japan) and the log-transformed coughs/h in 24 h at baseline as covariates. Modeled estimates are presented as percent change from baseline. Placebo-adjusted change is the percent change relative to placebo. *CI* confidence interval, *FAS* full analysis set

## **e-Table 8** Change from baseline in weekly cough severity by VAS at week 1, week 3, and week 4 (subgroup analysis: hourly cough count at Visit 2 ≥10 in the FAS)

| Modeled estimates^a^ | Sivopixant | | | Placebo |
| --- | --- | --- | --- | --- |
|  | 50 mg | 150 mg | 300 mg |  |
| Week 1 | n=84 | n=83 | n=83 | n=91 |
| Change, (95% CI) | −10.9 (−14.3, −7.5) | −11.2 (−14.7, −7.8) | −16.2 (−19.6, −12.7) | −10.7 (−14.0, −7.3) |
| Placebo-adjusted change, (95% CI) | −0.2 (−4.9, 4.5) | −0.6 (−5.3, 4.1) | −5.5 (−10.2, −0.8) | - |
| Week 3 | n=86 | n=82 | n=81 | n=86 |
| Change, (95% CI) | −19.6 (−24.1, −15.1) | −21.9 (−26.4, −17.4) | −28.5 (−33.1, −23.9) | −18.5 (−22.9, −14.0) |
| Placebo-adjusted change, (95% CI) | −1.1 (−7.4, 5.2) | −3.4 (−9.8, 2.9) | −10.0 (−16.4, −3.7) | - |
| Week 4 | n=83 | n=80 | n=81 | n=82 |
| Change, (95% CI) | −20.7 (−25.5, −15.9) | −21.6 (−26.4, −16.8) | −29.1 (−33.9, −24.2) | −20.9 (−25.7, −16.2) |
| Placebo-adjusted change, (95% CI) | 0.2 (−6.5, 6.9) | −0.7 (−7.4, 6.0) | −8.2 (−14.9, −1.4) | - |

^a^Differences are based on a mixed model for the change in weekly cough severity score after 4 weeks of treatment with treatment, week, and treatment-by-week as fixed effects; patient as random effect; and region (Europe, the United States, or Japan) and the severity score at baseline as covariates. *CI* confidence interval, *FAS* full analysis set, *VAS* visual analog scale

## **e-Table 9** Change from baseline in weekly cough severity by VAS at week 1, week 3, and week 4 (subgroup analysis: hourly cough count at Visit 2 <10 in the FAS)

| Modeled estimates^a^ | Sivopixant | | | | Placebo | |
| --- | --- | --- | --- | --- | --- | --- |
|  | 50 mg | 150 mg | 300 mg |  | |  |
| Week 1 | n=12 | n=12 | n=8 | n=10 | |  |
| Change, % (95% CI) | −9.8 (−21.3, 1.6) | −25.7 (−36.6, −14.8) | −11.3 (−24.8, 2.3) | −15.4 (−27.4, −3.4) | |  |
| Placebo-adjusted change, (95% CI) | 5.6 (−11.2, 22.4) | −10.3 (−26.5, 5.9) | 4.2 (−13.6, 21.9) | - | |  |
| Week 3 | n=11 | n=13 | n=10 | n=9 | |  |
| Change, % (95% CI) | −11.4 (−23.3, 0.4) | −31.8 (−42.8, −20.7) | −19.1 (−32.3, −5.9) | −25.1 (−37.4, −12.8) | |  |
| Placebo-adjusted change, (95% CI) | 13.6 (−3.6, 30.9) | −6.7 (−23.2, 9.8) | 6.0 (−11.7, 23.7) | - | |  |
| Week 4 | n=10 | n=12 | n=10 | n=11 | |  |
| Change, % (95% CI) | −13.6 (−26.5, −0.6) | −32.7 (−44.8, −20.6) | −21.2 (−35.4, −7.0) | −29.1 (−42.3, −16.0) | |  |
| Placebo-adjusted change, (95% CI) | 15.6 (−3.0, 34.2) | −3.6 (−21.4, 14.2) | 8.0 (−11.1, 27.0) | - | |  |

^a^Differences are based on a mixed model for the change in weekly cough severity score after 4 weeks of treatment with treatment, week, and treatment-by-week as fixed effects; patient as random effect; and region (Europe, the United States, or Japan) and the severity score at baseline as covariates. *CI* confidence interval, *FAS* full analysis set, *VAS* visual analog scale

## **e-Table 10** Change from baseline in weekly LCQ total score and PGIC after 4 weeks of treatment (subgroup analysis: hourly cough count at Visit 2 ≥10 in the FAS)

| LCQ - ≥1.3-point improvement in total score | | | | |
| --- | --- | --- | --- | --- |
| Response-level treatment | Responders  n/N (%)^a^ | Risk differences %  (95% CI)^b^ | Risk ratio  (95% CI)^b^ | Odds ratio  (95% CI)^b^ |
| Sivopixant 50 mg | 48/77 (62.3) | 3.9 (−12.3, 20.0) | 1.07 (0.82, 1.39) | 1.17 (0.62, 2.21) |
| Sivopixant 150 mg | 50/73 (68.5) | 10.8 (−4.8, 26.4) | 1.19 (0.92, 1.53) | 1.60 (0.81, 3.16) |
| Sivopixant 300 mg | 54/68 (79.4) | 20.7 (6.0, 35.4) | 1.35 (1.08, 1.69) | 2.82 (1.31, 6.07) |
| Placebo | 42/73 (57.5) | - | - | - |
| PGIC - any improvements | | | | |
| Response-level treatment | Responders  n/N (%)^a^ | Risk differences %  (95% CI)^b^ | Risk ratio  (95% CI)^b^ | Odds ratio  (95% CI)^b^ |
| Sivopixant 50 mg | 53/83 (63.9) | −6.1 (−20.8, 8.6) | 0.91 (0.73, 1.14) | 0.77 (0.40, 1.46) |
| Sivopixant 150 mg | 60/80 (75.0) | 5.6 (−8.3, 19.5) | 1.08 (0.89, 1.31) | 1.32 (0.66, 2.64) |
| Sivopixant 300 mg | 72/81 (88.9) | 18.7 (6.8, 30.7) | 1.27 (1.08, 1.49) | 3.59 (1.51, 8.53) |
| Placebo | 56/81 (69.1) | - | - | - |

^a^LCQ: Patients were considered responders if they met the improvement from the specified baseline threshold (≥1.3-points). For PGIC: Patients were considered responders if they reported “Very much improved,” “Much improved,” or “Minimally improved” on the PGIC assessment. ^b^Based on the Cochran-Mantel-Haenszel test stratified by region (Europe, the United States, or Japan) and cough count at baseline (≥30 coughs/h, <30 coughs/h) vs placebo. *CI* confidence interval, *FAS* full analysis set, *LCQ* Leicester Cough Questionnaire, *PGIC* Patient Global Impression of Change

## **e-Table 11** Change from baseline in weekly LCQ total score and PGIC after 4 weeks of treatment (subgroup analysis: hourly cough count at Visit 2 <10 in the FAS)

| LCQ - ≥1.3-point improvement in total score | | | | |
| --- | --- | --- | --- | --- |
| Response level-treatment | Responders  n/N (%)^a^ | Risk differences %  (95% CI)^b^ | Risk ratio  (95% CI)^b^ | Odds ratio  (95% CI)^b^ |
| Sivopixant 50 mg | 5/9 (55.6) | −13.0 (−61.2, 35.2) | 0.81 (0.39, 1.71) | 0.61 (0.09, 4.09) |
| Sivopixant 150 mg | 6/9 (66.7) | 0.0 (−46.2, 46.2) | 1.00 (0.50, 2.00) | 1.00 (0.16, 6.35) |
| Sivopixant 300 mg | 6/10 (60) | 3.4 (−44.6, 51.5) | 1.05 (0.54, 2.05) | 1.15 (0.17, 7.72) |
| Placebo | 6/9 (66.7) | - | - | - |
| PGIC - any improvements | | | | |
| Response level-treatment | Responders  n/N (%)^a^ | Risk differences %  (95% CI)^b^ | Risk ratio  (95% CI)^b^ | Odds ratio  (95% CI)^b^ |
| Sivopixant 50 mg | 4/10 (40) | −51.5 (−89.5, −13.5) | 0.43 (0.19, 1.00) | 0.10 (0.01, 0.97) |
| Sivopixant 150 mg | 12/12 (100) | 9.3 (−8.9, 27.5) | 1.10 (0.91, 1.34) | - |
| Sivopixant 300 mg | 7/10 (70) | −24.3 (−61.1, 12.5) | 0.73 (0.41, 1.30) | 0.25 (0.02, 2.58) |
| Placebo | 9/10 (90) | - | - | - |

^a^LCQ: Patients were considered responders if they met the improvement from the specified baseline threshold (≥1.3-points). For PGIC: Patients were considered responders if they reported “Very much improved,” “Much improved,” or “Minimally improved” on the PGIC assessment. ^b^Based on the Cochran-Mantel-Haenszel test stratified by region (Europe, the United States, or Japan) and cough count at baseline (≥30 coughs/h, <30 coughs/h) vs placebo. *CI* confidence interval, *FAS* full analysis set, *LCQ* Leicester Cough Questionnaire, *PGIC* Patient Global Impression of Change
